# Total Splenectomy due to an Unexpected “Complication” after Successful Extended Laparoscopic Partial Decapsulation of a Giant Epidermoid Splenic Cyst: A Case Report

**DOI:** 10.1155/2011/318208

**Published:** 2011-05-31

**Authors:** Michail Pitiakoudis, Petros Zezos, Anastasia Oikonomou, Prodromos Laftsidis, Georgios Kouklakis, Constantinos Simopoulos

**Affiliations:** ^1^2nd Department of Surgery, Democritus University of Thrace, University General Hospital of Alexandroupolis, 68100 Dragana, Alexandroupolis, Greece; ^2^Gastrointestinal Endoscopy Unit, Democritus University of Thrace, University General Hospital of Alexandroupolis, 68100 Dragana, Alexandroupolis, Greece; ^3^Department of Radiology, Democritus University of Thrace, University General Hospital of Alexandroupolis, 68100 Dragana, Alexandroupolis, Greece

## Abstract

Splenic cysts are rare entities and are classified as true cysts or pseudocysts based on the presence of an epithelial lining. Congenital nonparasitic true cysts can be epidermoid, dermoid, or endodermoid, present at a young age, and are commonly located in the upper pole of the spleen. Surgical treatment is recommended for symptomatic, large (more than 5 cm), or complicated cysts. Depending on cyst number, location, relation to hilus, and the major splenic vessels, the surgical options include aspiration, marsupialization, cystectomy, partial cystectomy (decapsulation), and partial or complete splenectomy. Laparoscopic techniques have now become the standard approach for many conditions, including the splenic cysts, with emphasis on the spleen-preserving minimally invasive operations. We present the successful extended partial laparoscopic decapsulation of a giant epidermoid splenic cyst in a young female patient that, although asymptomatic, was unfortunately followed by complete splenectomy five days later due to a misinterpreted abdominal CT suggesting splenic postoperative ischemia.

## 1. Introduction

Splenic cysts are rare entities and are classified as true cysts (primary) or pseudocysts (secondary) based on the presence of an epithelial lining. True cysts can be further subdivided into parasitic caused by *Echinococcus* and nonparasitic [1, 2].

Nonparasitic, true cysts are congenital or neoplastic. Congenital cysts can be epidermoid (90%), dermoid, or endodermoid, present at a young age (children and young adults) and are commonly located in the upper pole of the spleen. CA 19-9 and CEA levels are elevated in the epidermoid cyst's contents and in the patient's serum. Pseudocysts are believed to develop after posttraumatic intraparenchymal or subcapsular splenic hematomas and occasionally after splenic infarcts or infections. Secondary cysts account for 75% of all nonparasitic splenic cysts [1, 2].

The frequent use of abdominal imaging and the increasingly successful nonoperative management of splenic injuries contribute to a rise in the incidence of nonparasitic splenic cysts, which otherwise are considered to be rare.

Most of the patients with a splenic cyst have symptoms, and most asymptomatic cysts can be detected during physical examination of the abdomen [2]. Cysts larger than 5 cm are susceptible to hemorrhage, rupture, and infection. Surgical treatment is therefore recommended for symptomatic, large (more than 5 cm), or complicated cysts [2, 3].

Laparoscopic techniques have now become the standard approach for many conditions, including the splenic cysts, with emphasis on the spleen-preserving minimally invasive operations, since complete splenectomy carries the risk of overwhelming postoperative infection and thrombocytosis [4]. Apart from laparoscopic splenectomy, the laparoscopic spleen-preserving procedures for the treatment of splenic cysts depending on cyst number, location, relation to hilus, and the major splenic vessels include aspiration, marsupialization, cystectomy, partial cystectomy (decapsulation), and partial splenectomy [1, 2]. 

We report the successful extended partial laparoscopic decapsulation of a giant epidermoid splenic cyst in a young female patient that, although asymptomatic, was unfortunately followed by complete splenectomy five days later due to a misinterpreted abdominal CT suggesting splenic postoperative ischemia.

## 2. Case Presentation

A 19-year-old female patient was admitted at the emergency department in our hospital, complaining about abdominal pain in left upper quadrant. The patient's medical history was unremarkable, including previous abdominal trauma. During physical examination, a palpable mass was found in the left hypochondrium. The routine biochemical and hematological investigations were normal. 

**Figure 1:**
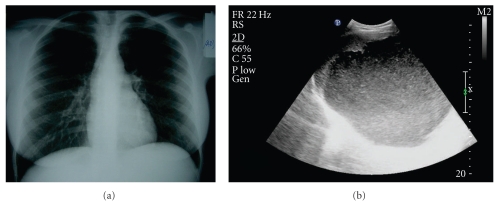
(a, b) Plain chest X-ray showing the elevation of the left hemidiaphragm (a). Abdominal ultrasound (US) showing a large, round hypoechoic lesion with internal echoes and regular thin wall surrounded by a peripheral rim of splenic tissue (b).

Chest and abdominal X-ray showed the elevation of the left hemidiaphragm (Figure 1(a)) and the abdominal ultrasound (US) revealed a large, round hypoechoic cystic lesion with internal echoes and regular thin wall surrounded by a peripheral rim of splenic tissue (Figure 1(b)). 

**Figure 2:**
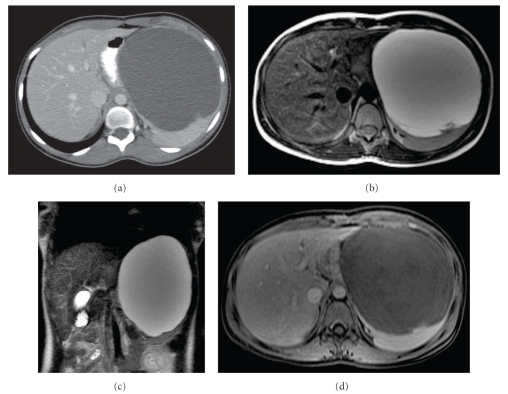
(a)–(d) Preoperative abdominal CT, (a) axial (b) and coronal (c) T2 weighted MRI shows a giant rounded cystic mass anterior to the spleen, which is significantly displaced posteriorly. Axial T1 weighted fat saturated MRI postgadolinium administration shows a thin peripheral enhancement of the splenic mass—possibly representing a capsule—and no internal enhancement consistent with its cystic nature (d).

The abdominal computed tomography scan (CT scan) demonstrated a well-circumscribed, large nonenhancing unilocular (17 cm × 12 cm × 15, 5 cm), hypoattenuated cystic (approximately 8 Hounsfield Units) lesion in the upper pole of the spleen lacking internal septa and therefore not favouring a parasitic origin (Figure 2(a)). The lesion produced displacement of the stomach and left liver lobe to the right, upward displacement of the diaphragm, and downward displacement of the left kidney. Pressure effects on the splenic vein and the body of pancreas were also demonstrated. An MRI study of the abdomen was performed including T1 weighted, T2 weighted, and STIR sequences followed by fast spoiled gradient echo 3D (T1 FAME) fat saturated pre- and postgadolinium enhanced images on axial and coronal planes. A giant well-defined cystic mass of low T1 and high T2 signal intensity images was detected. There was a thin, mild peripheral enhancement possibly representing a capsule, and there was no enhancement centrally after intravenous administration of gadolinium (Figures 2(b), 2(c), and 2(d)).

Serum levels of CA 19-9 were elevated (132; normal value <40 U/mL), while serum levels of CEA were normal and Echinococcus serum antibodies were negative.

The negative history for antecedent abdominal trauma together with the imaging and laboratory findings were suggestive of a giant symptomatic primary nonparasitic congenital splenic cyst for which surgical treatment was indicated.

**Figure 3:**
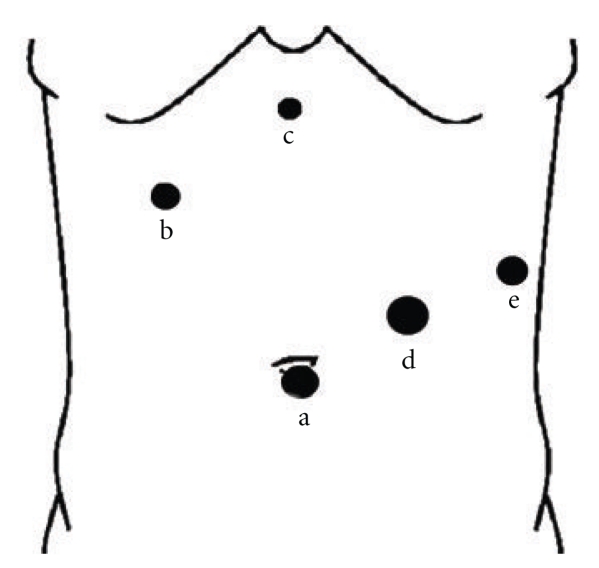
Port positions. A 10-mm umbilical port for laparoscopy (a), a 10 mm on the right upper quadrant below the right costal margin on the midclavicular line (b), a 5 mm trocar in the sub-xiphoid region (c), a 12 mm trocar paraumbilical in the left mid-clavicular line (d) and a 10 mm trocar in the left midaxillary line (e).

Surgical treatment was carried out by laparoscopic approach. Under general endotracheal anesthesia, the patient was placed in a modified lithotomy 20° antitrendelenburg position with right lateral position at 10°. A 10-mm trocar was inserted in a standard umbilical position using the Hasson's open technique, pneumoperitoneum was created by carbon dioxide insufflation, and a 30° camera was inserted subsequently. Due to the large size of the cyst that caused displacement of adjacent organs and structures, additional trocars were placed as shown in Figure 3: one 12 mm trocar paraumbilical in the left midclavicular line, one 10 mm trocar in the left midaxillary line, one 5 mm trocar in the subxiphoid region and another 10 mm on the right upper quadrant on the midclavicular line. 

**Figure 4:**
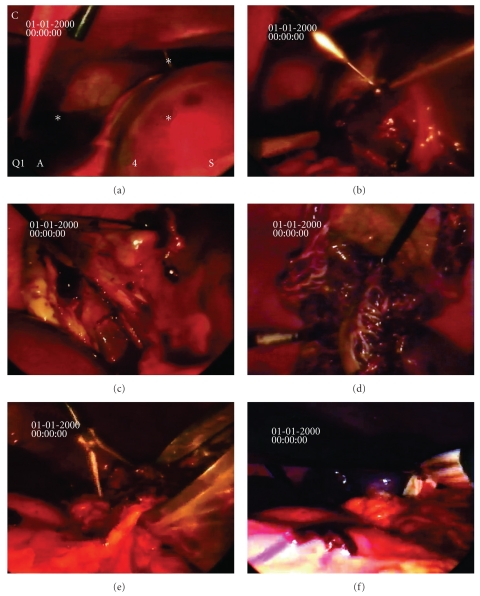
(a)–(f) Laparoscopic view of the splenic cyst during operation. A large splenic cyst located at the upper pole of the spleen was found, and its contents were aspirated laparoscopically (a & b). Laparoscopic resection of the cyst wall and partial decapsulation with a large window was performed (c & d). The resected cyst wall was removed in an ENDOBAG (e). A drain was placed in the cyst remaining cavity (f).

The omentum attached over the spleen was dissected and pulled down revealing an anterior cyst at the upper pole of the spleen (Figure 4(a)). The splenic flexure of the colon was mobilized, and the splenocolic ligament was divided with LigaSure 5 mm vessel sealing system. Furthermore, the splenophrenic attachments and the gastrosplenic ligament were dissected and divided, and the short gastric vessels were ligated. Afterwards, with the same precautions as when dealing with a parasitic cyst, the cyst was punctured with a needle at the most protruding area (Figure 4(b)). Serous fluid was partially evacuated, and lavage of the cyst cavity was performed with hypertonic saline. The aspirated cystic contents showed epithelial fragments with no evidence of malignancy, and biochemical analysis showed high levels of CA19-9. Using the LigaSure vessel sealing system, cyst wall was almost completely resected except for the wall adjoining to the residual splenic parenchyma, with excellent hemostasis while respecting the lower pole of the spleen (Figures 4(c) and 4(d)). Afterwards, the resected cyst capsule was removed with an ENDO CATCH bag (Figure 4(e)), and a drain was placed in the left subphrenic space with the tip in the remaining cystic cavity (Figure 4(f)). The procedure was carried out in 135 minutes with minimal blood loss (120 mL). 

Histologic examination of the resected specimen showed that it was a 9 cm in diameter part of a splenic cyst. The inner surface had septations and multiple trabeculations, which were gray yellow and firm. The cyst wall contained hyalinizated dense fibrous tissue, and the cortex was covered with keratinized squamous epithelium. The lining varied from flattened single-layer cuboidal to fully stratified keratinized squamous epithelium. There was no evidence of epithelial atypia or malignancy, the stratified squamous epithelium was positive in the immunohistological stains for CA19-9, ceratines 8, 18, and 19. The histopathological findings confirmed the diagnosis of a congenital splenic epidermoid cyst. 

**Figure 5:**
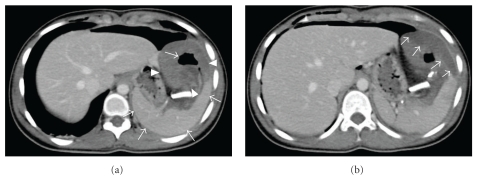
(a, b) Axial enhanced CT postsurgical decapsulation of the splenic cyst (3rd post-operative day) reveals an intact and homogeneously enhanced splenic parenchyma (a). The hypodense area at the anatomic region of the decapsulated cyst anterior to the spleen (a) that contains loculation of air in its center (a) represents the remnants of the cystic wall that have partially collapsed after laparoscopic surgical decapsulation together with perisplenic pockets of fluid (b).

**Figure 6:**
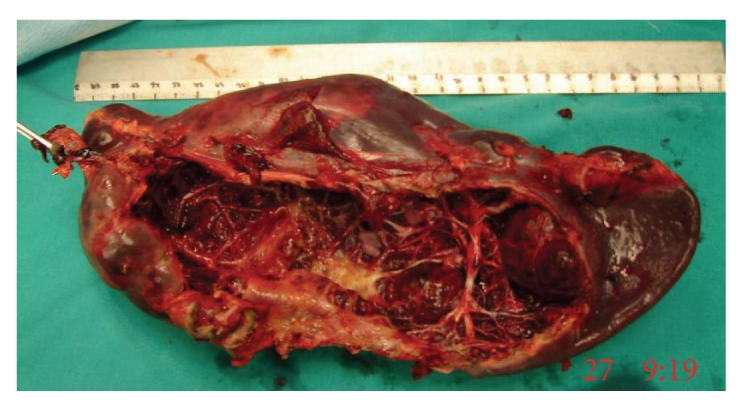
The surgical specimen after total splenectomy containing the spleen and the cystic remnant. The trabecular architecture characteristic of a nonparasitic congenital splenic cyst and the intact spleen are clearly seen.

The patient's recovery was prompt and clinically uneventful. A follow-up abdominal CT scan at the 3rd postoperative day showed perisplenic fluid accumulation and an air-filled cavity contiguous with the splenic parenchyma, while the parenchyma of the splenic remnant was falsely misinterpreted as having perfusion defects, especially in the margins of the cystic wall left in situ (Figures 5(a) and 5(b)). Subsequently, the asymptomatic patient underwent a total splenectomy for possible splenic ischemia according to CT findings which were suggestive of perioperative vascular injury. An Endo-GIA linear stapler was applied 2 times to accomplish total splenectomy along the splenic hilum, avoiding injuries to the distal pancreas. The intact resected spleen was retrieved (Figure 6), and a drain was placed in the left subphrenic space, and all incisions were closed in the usual way. The operation took about 45 min with no blood loss.

The histologic examination of the resected specimen (23 cm × 11 cm × 3.5 cm, weight 548 gr) revealed a macroscopically intact spleen together with its portal vessels and a cystic wall remnant. The splenic parenchyma presented mild congestion and edema without evidence of ischemia or infarction. The cystic wall attached to the splenic remnant had the same histologic features with the previously resected cyst. 

The postoperative course was uneventful, and the patient was vaccinated against postsplenectomy infections and discharged 7 days later with a normal abdominal US and Doppler exanimation. The platelet count increased to 850000/mm^3^ at 10 days, but returned to within the normal range by 3 weeks after surgery. Her serum levels of CA19-9 were normal 4 months after the operation. There have been no abdominal symptoms during 12 months of follow-up.

## 3. Discussion

Nonparasitic cysts larger than 5 cm are susceptible to hemorrhage, rupture, and infection. Therefore, surgical treatment is recommended for symptomatic, large (more than 5 cm), or complicated cysts [2]. The conventional treatment of nonparasitic splenic cysts has been total splenectomy, open or laparoscopic. In recent years, a spleen-preserving surgical approach is recommended when treating splenic diseases since it is well known that the spleen plays an important role in normal homeostasis. Spleen is involved in several functions including the regulation of the circulating blood volume, hematopoiesis, immunity, and protection against infections and malignancies. Postsplenectomy overwhelming infection and sepsis are serious morbid complications with high fatality rate [1, 2]. 

Laparoscopic techniques have now become the standard approach for many conditions, including the splenic cysts, with emphasis on the spleen-preserving minimally invasive operations [2]. Laparoscopic techniques for nonparasitic splenic cysts include aspiration, marsupialization, fenestration, partial cystectomy (decapsulation), and laparoscopic partial splenectomy with the resection of the cyst and a portion of the contiguous splenic parenchyma.

A high proportion of cyst recurrence has been reported after simple aspiration or marsupialization of the cyst, and, therefore, these techniques are not recommended [1, 2]. The definite treatment of the cyst without recurrence is achieved after complete removal of the cyst wall and lining in its entirety. Morgenstern and Shapiro reported the first resection of the cyst and a portion of the contiguous splenic parenchyma (partial splenectomy) [5], while later on the next decades laparoscopic partial splenectomy for nonparasitic splenic cysts has been reported [6–8]. There are no reports of recurrent cysts after partial splenic resection [9].

When partial splenic resection is not feasible due to technical difficulties, some authors have proposed an alternative technique called “cystic decapsulation”. Decapsulation involves a near total resection of the cyst leaving the portion of cyst wall contiguous with the splenic parenchyma in situ. Decapsulation is a simpler and more rapidly performed procedure, with less blood loss than partial splenectomy [10–13]. The disadvantage of this technique is the possibility of cyst recurrence because a portion of the cyst lining has been left intact [14]. However, the recurrence rate is reported in a smaller proportion compared to aspiration or marsupialization and depends on the extent of the incomplete cyst wall removal. Salky et al. [15] described the first laparoscopic partial cystectomy with decapsulation that showed no signs of recurrence after five years of observation, while Touloukian et al. [16, 17] reported the successful open cystic decapsulation in adult and pediatric patients.

Finally, although perioperative complications of the laparoscopic treatment for splenic diseases have been reported [2], open procedures carry a higher complication risk and a higher median postoperative hospital stay [4].

In our case, the female patient presented with a large symptomatic splenic cyst. Laboratory and imaging studies were suggestive of a primary nonparasitic congenital splenic cyst, and surgical treatment was decided as recommended [1, 2]. We decided to treat the cyst with a spleen-preserving laparoscopic method performing extended partial decapsulation of the cyst as it has been previously described. Unfortunately, although the decapsulation was successful without overt intra- or postoperative complications, we performed a total splenectomy in the 3rd postoperative day since splenic ischemia was falsely suspected in a postoperative follow-up abdominal CT. 

Iatrogenic splenic injury is a rare complication and may be caused during abdominal surgery, cardiac surgery, and colonoscopy [18, 19]. Splenic injury includes contusion, laceration, hematoma, active hemorrhage, posttraumatic pseudoanerysm, and posttraumatic infarction. Surgical procedures, open or laparoscopic, involving organs in the left upper abdomen, are the most common causes of splenic injury. Most intraoperative injuries are promptly detected and treated during operation conservatively or by splenectomy [18, 19]. Contrast-enhanced CT is the diagnostic imaging tool of choice for the detection of possible splenic injury in patients with complicated postoperative course. Additionally, contrast-enhanced CT features of splenic injury serve in guiding the treatment [20, 21].

Splenic infarction may develop insidiously during surgery and may be diffuse or focal. Peripheral splenic arterial branches are end arteries with poor collateral circulation. Perfusion defects in these vessels after occlusion, injury, or severe hemodynamic derangement during operation leads to infarction. Causes of splenic artery occlusion include embolic disease (e.g., in mitral valve disease), atherosclerosis, arteritis, splenic artery aneurysm, sickle cell disease (thrombosis), splenic torsion, and mass lesions such as pancreatic carcinoma [21, 22].

 Splenic infarcts may be silent or the patients may present left upper quadrant pain of acute onset with or without fever. The CT appearance of splenic infarction is time depended after the triggering event [22]. The infarcts are usually wedge-shaped areas of nonenhancement but may also be irregular, and they may be single or multiple. The infarct may completely disappear or result in a residual contour defect representing scarring. Usually small, uncomplicated splenic infarcts are conservatively managed, while complicated or those indicating severe vascular compromise of the spleen affecting its viability are treated with complete splenectomy [21]. 

In the present case, an iatrogenic intraoperative injury during recent laparoscopic decapsulation or postoperative torsion of the vessels at the splenic hilum due to extended mobilization was considered as the possible causes of splenic ischemia due to the CT findings in an otherwise asymptomatic patient. Total splenectomy was consequently performed, while the histologic examination of the resected spleen showed an intact splenic parenchyma with mild congestion and edema without evidence of ischemia or infarction. A thorough search of the literature in PubMed did not reveal any report of a similar complication after laparoscopic cyst decapsulation of the spleen. In a single case, as reported by Chin et al., splenic ischemia was recognized during laparoscopic partial splenectomy and was treated with conversion to total splenectomy [13]. 

In this case we showed the CT imaging appearance of a normal spleen in the 3rd postoperative days after successful and uneventful laparoscopic partial cyst decapsulation. We did not find a similar image in the literature, except in one case of a spontaneously ruptured congenital splenic cyst, which had a CT appearance resembling to our case [23]. Retrospectively, we believe that the nonenhancing areas of splenic parenchyma in the vicinity of the splenic cyst wall were not consistent with perfusion defects but represented pockets of perisplenic fluid. With this case, we underscore the significance of the postoperative contrast-enhanced CT examination, which is the method of choice in detecting possible iatrogenic splenic injuries and in affecting decision making for their management. Therefore, close team work between the radiologist and the surgeon is highly recommended in order to set the diagnosis of possible postoperative complications or assure spleen-preserved operations.

## 4. Conclusion

The laparoscopic partial cyst decapsulation is a safe and feasible surgical approach for the treatment of large congenital splenic cysts offering the advantages of a simple, not time-consuming, spleen-preserving technique with minimal blood loss, no major complications and short postoperative recovery. Contrast-enhanced CT is the diagnostic imaging modality of choice for the detection of possible splenic injury in patients with complicated postoperative course. The critical consideration of clinical signs together with imaging finding must be taken to account for the optimal treatment of a splenic injury iatrogenic or not, avoiding thus unnecessary splenectomies.
